# Impact of an educational program on community pharmacist’s preparedness to conduct pharmacist-led diabetes clinic in Saudi Arabia

**DOI:** 10.1186/s40545-023-00597-2

**Published:** 2023-07-13

**Authors:** Muhammad Kamran Rasheed, Syed Shahzad Hasan, Abdulmajeed Alqasoumi, Zaheer Ud-Din Babar

**Affiliations:** 1 Department of Pharmacy Practice, College of Pharmacy, Qassim University, Buraydah, Saudi Arabia; 2 School of Pharmacy, University of Huddersfield, Huddersfield, HD1 3DH UK

**Keywords:** Diabetes care, Diabetes mellitus, Community pharmacist, Public health, Education, Attitude to health

## Abstract

**Background:**

Demand for diabetes care and prevention has increased due to Saudi Arabia’s high prevalence of diabetes mellitus and its insufficient treatment. This raised awareness of the significance of community pharmacists in Saudi Arabia, who may significantly improve diabetes treatment by setting up pharmacist-led diabetic clinics. Thus, to assess community pharmacists’ readiness to lead diabetes clinics in Saudi Arabia, this study evaluated the usefulness of an educational session on diabetes care.

**Method:**

The preparation of community pharmacists for diabetes treatment and management was assessed using a validated diabetes-specific questionnaire. An engaging and thorough diabetes education class was presented by two licensed diabetes educators. One-way ANOVA, chi-square, and the Mann–Whitney U-test were used to statistically assess the pre- and post-knowledge and attitude scores of community pharmacists.

**Results:**

Following a learning session, the community pharmacists had a significant increase in understanding oral hypoglycemic medicines, monitoring the disease’s course, and dosing of insulin for diabetics *(p* = *0.01).* Additionally, the community pharmacist’s perspective and attitude score on managing diabetes increased from 49.74 to 52.74 *(p* = *0.01).*

**Conclusion:**

The study’s findings demonstrated a marked improvement in community pharmacist’s knowledge of and attitude toward running pharmacist-led diabetic clinics following a session on diabetes education in collaboration with the Pharmacy College. The study’s findings also emphasized the significance of developing a structured programme for diabetes education in Saudi Arabia to address the demands of community pharmacists in terms of professional development.

## Introduction

With a 2.2% yearly growth rate, Saudi Arabia has one of the highest per capita prevalence rates of diabetes mellitus (DM) in the world (23.7% of the population) [1]. Healthcare facilities are addressing the nation’s escalating cases of diabetes-related microvascular and macrovascular problems [1, 2]. One of the causes for the nation’s insufficient management of diabetes and its consequences is a lack of understanding of the risk factors for diabetes among the general population and inadequate glycaemic management by healthcare professionals [3]. Additionally, as patients’ demands for diabetes prevention and monitoring have increased as a result of social media awareness of lifestyle choices, the strain on healthcare practitioners to schedule more outpatient sessions and respond to patient concerns and questions has increased [1, 4].

Patient-centred diabetes care provided by community pharmacists helps prevent and manage diabetes-related problems and aids in achieving patient-centric therapeutic, financial, and humanistic objectives [5–7]. Improvements in glycaemic control, medication adherence, and HbA1C have been seen in diabetic patients under community pharmacist-managed care [6, 8].

To achieve therapeutic effects through collaborative decision-making, monitoring, and administration of diabetic treatment, Saudi Arabia’s easily accessible community pharmacy can be an appropriate health destination [4, 9]. However, community pharmacists should possess current clinical knowledge about the management and monitoring of diabetes mellitus and a professional and empathetic attitude to deliver effective diabetes care to patients [3, 10].

Continuing professional development programmes provided by professional organisations have resulted in the integration of information, abilities, and attitudes among community pharmacists, which may be used to improve patient care and management [11]. Currently, there is no formal and structured continuing professional development and training programme for community pharmacists offered by pharmacy colleges or other professional healthcare organisations in Saudi Arabia [10, 12]. Community pharmacists in Saudi Arabia have expressed an interest in skill development and educational programmes to enable them to provide pharmacist-led clinics and specialised treatment to patients [10]. Although many studies have demonstrated improvements in community pharmacists’ understanding, attitudes, and capacities to deliver efficient patient-centred care following an educational and training session, none have discovered a connection between a structured diabetes training programme offered in collaboration with a Pharmacy College in Saudi Arabia and community pharmacists’ preparedness to establish an effective diabetes clinic. Therefore, the purpose of this study is to assess, design, and carry out an educational session to assist community pharmacists in Saudi Arabia to be more prepared to deliver patient-centred diabetes care services.

## Methods

### Study design

A 1-day educational session on patient-centered diabetes care and management was conducted in collaboration with the College of Pharmacy, Qassim University of Saudi Arabia. The research participants were licenced and practising community pharmacists. The learning materials and presentation of the lectures on diabetes education sessions were made by two clinical pharmacists, one of whom had advanced certification in diabetes care from the United States. After going thorough extensive literature research on the most recent diabetes guidelines and local Saudi patient’s preferences and challenges, the educational materials were developed. Table 1 lists the 5 lessons and 22 sub-topics for the educational session. Each lecture was presented as a PowerPoint presentation and followed by patient case discussions and a question-and-answer session.

**Table 1:** The distribution of lessons, contents, and time allocation of the diabetes care educational session

Topics and format	Contents	Time allocation in hours
1.** Lectures**		
Introduction to diabetes care	Introduction to diabetes, risk factors of developing diabetes, normal blood glucose and HbA1c values, pre-diabetes evaluations and management of micro and macrovascular complications	1.5
Therapeutic goals in diabetes mellitus	What is a glycaemic index, what are nutritional goals in diabetes mellitus and lifestyle interventions to manage diabetes care	1
Role of oral hypoglycaemic agents and insulins in diabetes management	Use of metformin, incretins and sodium–glucose cotransporter-2 inhibitors and thiazolidinediones	2
Diabetes care in special population	Management of hypoglycaemia and different types of insulins	
	Using basal and mealtime insulins and avoiding diabetic ketoacidosis	
	Diabetes management in children, pregnant women and the elderly, diabetic retinopathy and neuropathy and diabetic foot care management and prevention	2
2. **Case discussion and question–answer session**	Discussion of diabetic cases, identifying drug-related problems and recommendations for a patient-centric approach	1.5
Total		8

All regional managers of chain pharmacy groups were emailed programme material and invitations for approval and subsequent distribution to their community pharmacist’s network via official WhatsApp or other social media groups. Some invites were physically delivered to community pharmacists, particularly those working in independent pharmacies, by visiting their workplaces.

### Instrument development

The demographics of the research participants are covered in the first section of the questionnaire (Table 2).

**Table 2:** Demographic distribution of community pharmacists attending the educational session on diabetes care

Demographic characteristics	Frequency N (%)
Gender	N = 40
Male	35 (87.5)
Female	5 (12.5)
Nationality	N = 40
Egyptian	30 (75)
Saudi	6 (15)
Indian	3 (7.5)
Sudani	1 (2.5)
Age group	N = 40
25–30	21 (52.5)
31–40	18 (45)
More than 40	1 (2.5)
Year of experience overall	N = 40
Less than a year	5 (12.5)
1–3 years	3 (7.5)
3–6 years	12 (30)
6–10 years	14 (35)
More than 10 years	6 (15)
Qualification	N = 40
Bachelors in pharmacy	33 (82.5)
Doctor of pharmacy	6 (15)
Master of pharmacy	1 (2.5)
Years of community pharmacy practice in Saudi Arabia	N = 40
0–1 years	7 (17.5)
1–3 years	5 (12.5)
3–6 years	13 (32.5)
6–10 years	12 (30)
More than 10 years	3 (7.5)

#### Knowledge test

To evaluate community pharmacists’ knowledge of diabetes care, a modified version of the University of Michigan Diabetes Research and Training Center (MDRTC) Diabetes Knowledge Test (DKT) was employed [13, 14]. The modified version of the DKT consists of four subcategories of 17 multiple-choice questions (Table 3). Each accurate response earns a research participant a score of “1”, whereas every wrong response earns them a score of “0”.

**Table 3:** Comparison of the knowledge and attitude scores of community pharmacists before and after the educational session on diabetes care

Categories	Items	Pre-test mean (± SD)	Post-test mean (± SD)	*p-value*
Knowledge				
Nutrition and monitoring	7	4.13 ± 1.14	5.45 ± 0.97	< 0.001
Oral pharmacotherapy	3	2.17 ± 0.92	2.65 ± 0.57	0.003
Knowledge about insulin	4	2.25 ± 0.86	3.05 ± 0.8	< 0.001
Complications of diabetes	3	2.47 ± 0.74	2.9 ± 0.3	< 0.001
Total knowledge scores	17	11.02 ± 0.79	14.05 ± 1.12	0.01
Attitude				
Special training in diabetes	4	17.72 ± 2.29	18.6 ± 1.53	0.02
Complications in diabetes	3	11.4 ± 2.22	12.22 ± 2.26	0.04
Value of tight control	2	8.85 ± 1.15	9.18 ± 0.92	0.07
Patient autonomy	3	11.77 ± 1.94	12.92 ± 1.72	0.003
Total attitude scores	12	49.79 ± 3.25	52.91 ± 3.4	0.01

#### Attitude test

To evaluate community pharmacist’s attitudes about managing and monitoring diabetes and its consequences, a modified version of the Diabetes Attitude Scale (DAS-3) was developed [15] (Table 3). The research participants were asked to rate statements on a 5-point Likert scale of the DAS-3 questionnaire at both time points. From the 12 attitude questions of the DAS-3 scale, Questions 1–4, 8, and 10–12 have scores ranging from “5” for strong agreement and “1” for strong disagreement. For Questions 5–7 and 9, a reverse scoring method is used to minimise recall bias. Higher attitude scores indicate a more positive attitude of community pharmacists toward diabetes care.

Although both DKT and DAS-3 scales were already validated and pre-tested, their modified local version was again tested for reliability and suitability by an expert panel consisting of a diabetologist, a clinical pharmacist and an expert academic and researcher in survey-based questionnaire development. Ten community pharmacists evaluated the questionnaire’s pilot testing to check for usability, flow, layout, and clarity of the question stems. Following comments from community pharmacists who weren't included in the study, a few minor adjustments were made to the questionnaire to make it easier to complete.

### Data collection

Before the lectures, all research participants received a briefing about the study objectives and aims and how to fill out the questionnaire. Furthermore, the questionnaires were distributed to all research participants before and after the educational session. The distribution of the questionnaire was aided by a group of six pharmacy interns. To assess the success of the diabetes education session, community pharmacists’ pre- and post-test knowledge and attitude scores were compared. The ethical permission was given by Qassim University’s ethics and scientific research committee (R 19/06/2019). In addition, consent has been taken from all participating community pharmacists, who were also informed that their participation was voluntary, and anonymous and all their data would be kept private and confidential.

### Analysis of the data

To determine the study population of the community pharmacists, Epi Info online software was employed [16]. The programme estimated a sample size of 52 community pharmacists with a 95% confidence interval and a 5% allowable margin of error. Nevertheless, 40 community pharmacists showed up for the educational session, representing a response rate of 77%.

SPSS, version 26 for Windows, was used for descriptive and inferential statistical analysis. Community pharmacist demographics, frequencies, and percentages were used for categorical variables in the descriptive analysis. The practice and knowledge scores were assessed using the mean (SD) or frequency (%). The student t-test, one-way ANOVA and the chi-square tests were used to assess the differences between the attitude and knowledge scores of community pharmacists before and after the diabetes educational session. A p ≤ 0.05 value was deemed significant by the statistical analysis. A total score of 17 is the maximum possible score for the DKT scale, while a score of 60 was considered the best attitude toward diabetes care on the DAS-3 scale. The Mann–Whitney U-test was used to compare demographic data with changes in the knowledge and attitude scores of research participants.

## Results

Details of the research participants can be found in Table 2. Among the forty community pharmacists attending the diabetes educational session, the majority were male (35, 87.5%). Nevertheless, five female community pharmacists, all fresh Pharm D graduates, having an overall experience of less than 1 year also attended the educational session (5, 12.5%). In contrast, the majority of males (30, 75%) were non-Saudis with a B. Pharm degree. Most community pharmacists had a total work experience ranging from 6 to 10 years (14, 35%).

### Community pharmacist’s preparedness to conduct diabetes care clinic

To evaluate the community pharmacist’s clinical knowledge to conduct diabetes clinic, Table 3 compares mean knowledge scores before and after the diabetes training session. When we compared the knowledge scores of community pharmacists before and after the educational session, it showed significant differences in all four categories of the DKT scale (Table 3) (*p* < *0.05*). Following the educational session, community pharmacists’ knowledge of managing diet in diabetes mellitus and monitoring the condition significantly improved *(p* = *0.001).* The knowledge of community pharmacists regarding the use of oral hypoglycaemic medications in the management of diabetes mellitus, however, showed the least improvement.

Table 4 shows that compared to male community pharmacists (9.97 ± 2.76)., females had statistically better mean pre-intervention scores (10.5 ± 1.29). The pre-intervention scores of community pharmacists with less than 3 years of experience was overall were higher (11.5 ± 1) than those with more than 3 years of experience.

**Table 4:** Demographic comparison of the change in the knowledge and attitude scores of community pharmacists before and after the diabetes care educational session

Variable	Change in knowledge			Change in attitude		
	Pre-test mean ± SD	Post-test mean ± SD	p-value	Pre-test mean ± SD	Post-test mean ± SD	p-value
Gender						
Male	9.97 ± 2.76	14.75 ± 2.15	0.985	49.19 ± 5.89	51.55 ± 4.91	0.492
Female	10.5 ± 1.29	15.25 ± 0.95		52.25 ± 5.18	54.75 ± 4.57	
Age						
25–30	10.10 ± 2.31	14.55 ± 2.48	0.722	51.1 ± 4.45	52.6 ± 4.28	0.972
31–40	10.00 ± 3.07	15.16 ± 1.53		48.21 ± 6.72	51.57 ± 5.30	
More than 40	9.00 ± 0.1	13.00 ± 0.1		42	43	
Total years of experience						
Less than 1 year	11.00 ± 1.58	15.60 ± 1.14	0.998	51 ± 5.29	55.2 ± 4.08	0.289
1–3 years	11.00 ± 1.41	16.00 ± 1.41		48 ± 8.48	48 ± 4.24	
3–6 years	9.67 ± 2.57	14.67 ± 1.87		50.16 ± 4.01	53 ± 2.93	
6–10 years	9.75 ± 3.41	14.44 ± 2.60		48.12 ± 6.86	50.23 ± 4.96	
More than 10 years	10.40 ± 0.89	15.00 ± 1.58		51.4 ± 6.94	53.2 ± 7.53	
Education						
Bachelors in pharmacy	9.88 ± 2.79	14.68 ± 2.18	0.998	49.5 ± 5.9	51.41 ± 4.97	0.885
Doctor of pharmacy	11.00 ± 1.58	15.60 ± 1.14		51 ± 5.29	55.2 ± 4.08	
Master of pharmacy	10.00 ± 0.01	15.00 ± 0.01		42	51	
Years of practice in community pharmacy in Saudi Arabia						
Less than 1 year	10.14 ± 2.61	14.86 ± 1.95	0.957	49.71 ± 4.92	54 ± 4.08	0.498
1–3 years	11.50 ± 1.00	16.00 ± 1.15		50 ± 5.47	51.25 ± 4.92	
3–6 years	9.53 ± 2.26	14.00 ± 2.44		49.17 ± 7.15	51.76 ± 4.39	
6–10 years	10.22 ± 4.05	15.56 ± 1.23		49.11 ± 4.04	51.77 ± 6.39	
More than 10 years	10.00 ± 1.00	15.33 ± 2.08		51.33 ± 8.14	48.66 ± 6.02	

Community pharmacists’ overall attitudes about managing diabetes have improved, going from a pre-intervention score of 49.74 ± 3.25 to a post-intervention score of 52.74 ± 3.42 *(p* = *0.01)*. Table 3 shows that the community pharmacist’s attitude toward specialized training in diabetes management *(p* = *0.02),* preventing microvascular and macrovascular complications of diabetes mellitus *(p* = *0.04),* and securing patient’s privacy and rights *(p* = *0.003)* showed noteworthy improvements after the diabetic educational session. However, the attitude of community pharmacists regarding diabetes monitoring did not alter considerably *(p* = *0.07).*

Statistically, no notable variations have been noticed across different age groups, gender, educational degree, and years of experience on the pre-and post-knowledge and attitudes scores of the community pharmacists (Table 4). Even though most knowledge and attitude scores were reported to be higher following the educational intervention, three research participants with a combined experience of 1–3 years in community pharmacy did not exhibit any improvement in their attitude. Similarly, three research participants with experience of more than 10 years in community pharmacy practice demonstrated negative improvement in attitude following an instructional session (51.33 ± 8.14 vs 48.66 ± 6.02).

## Discussion

According to the study’s findings, community pharmacists can successfully conduct pharmacist-led diabetes clinics in community pharmacy settings across Saudi Arabia if provided a structured and targeted diabetes education programme delivered by certified diabetes educators in collaboration with professional pharmacy organizations. The study found that community pharmacists’ knowledge and attitudes toward diabetes care and management improved significantly following the educational session. Similar outcomes have been reported in Croatia and India, where the clinical expertise of community pharmacists increased following an interactive clinical pharmacy workshop [17, 18].

The findings of this pre- and post-educational intervention study are also consistent with previously published studies from around the world, which demonstrated that skills and knowledge-enhancing programmes can significantly keep community pharmacists up to date on the latest guidelines and skills required for patient-centred pharmaceutical care services and to establish result oriented pharmacist-led clinics [5, 19, 20].

The Saudi Commission of Health Specialties (SCFHS) requires all pharmacists to earn 40 CPD points within 2 years to renew their practice license. The SCFHS standards for CPD points, on the other hand, are not structured and do not encourage patient-centred care practices at the community pharmacy level. In addition, Saudi Arabia does not have a separate pharmacy board representing pharmacists at the national level [21–23]. In contrast, attending a CPD program in the Western world is well-established and structured and mainly provided through national pharmacy boards [24, 25].

The study’s findings support the idea that Saudi Arabia needs a comprehensive, standardized, and structured diabetes education programme offered through professional pharmacy organizations. These programs could enhance the community pharmacists’ clinical knowledge and skills and improve their ability to provide effective diabetes care and monitoring through pharmacist-led diabetes clinics.

The authors of this study believe that the methodology used in this study and the findings may pave the way for future studies in Saudi Arabia that can quantify the long-term impact of a structured diabetes education program on community pharmacist’s preparedness to conduct effective diabetes clinics.

This pre-and post-educational intervention study to assess community pharmacist’s knowledge and attitudes toward diabetes care and management is the first of its kind to be carried out in Saudi Arabia or elsewhere in the Middle East, as far as the authors are aware.

The sample size of community pharmacists in this study may not reflect the knowledge and attitudes of all community pharmacists in the country, which could be its limitation. The long-term impact of an educational session on different time scales will be required in future research to assess the preparedness of community pharmacist’s in conducting effective diabetes clinics at community pharmacy settings in Saudi Arabia.

## Conclusion

The study findings supported that community pharmacists in Saudi Arabia could contribute toward the prevention and management of diabetes mellitus by establishing pharmacist-led diabetes clinics, provided they get structured professional training from certified diabetes educators. The study’s findings underlined the importance of a countrywide structured training and skills development programme given by professional pharmacy organisations in increasing community pharmacists’ diabetes knowledge and patient-centred attitude.

## Data Availability

Not applicable.
